# Potential of Kale and Lettuce Residues as Natural Adsorbents of the Carcinogen Aflatoxin B_1_ in a Dynamic Gastrointestinal Tract-Simulated Model

**DOI:** 10.3390/toxins13110771

**Published:** 2021-10-31

**Authors:** Alma Vázquez-Durán, María de Jesús Nava-Ramírez, Daniel Hernández-Patlán, Bruno Solís-Cruz, Víctor Hernández-Gómez, Guillermo Téllez-Isaías, Abraham Méndez-Albores

**Affiliations:** 1 Unidad de Investigación Multidisciplinaria (UIM) L14 (Alimentos, Micotoxinas, y Micotoxicosis), Facultad de Estudios Superiores Cuautitlán (FES-C), Universidad Nacional Autónoma de México (UNAM), Mexico City 54714, Mexico; almavazquez@comunidad.unam.mx (A.V.-D.); mari_551293@comunidad.unam.mx (M.d.J.N.-R.); 2 UIM L5 (LEDEFAR), FES-C, UNAM, Mexico City 54714, Mexico; danielpatlan@comunidad.unam.mx (D.H.-P.); bruno_sc@comunidad.unam.mx (B.S.-C.); 3 UIM LB (Investigación en Energías Renovables), FES-C, UNAM, Mexico City 54714, Mexico; vichugo@unam.mx; 4 Department of Poultry Science, University of Arkansas, Fayetteville, AR 72701, USA; gtellez@uark.edu

**Keywords:** aflatoxin B_1_, agro-waste-based sorbents, in vitro digestion model

## Abstract

Adsorption of the carcinogen aflatoxin B_1_ (AFB_1_) onto agro-waste-based materials is a promising alternative over conventional inorganic binders. In the current study, two unmodified adsorbents were eco-friendly prepared from kale and lettuce agro-wastes. A dynamic gastrointestinal tract-simulated model was utilized to evaluate the removal efficiency of the sorptive materials (0.5%, *w/w*) when added to an AFB_1_-contaminated diet (100 µg AFB_1_/kg). Different characterization methodologies were employed to understand the interaction mechanisms between the AFB_1_ molecule and the biosorbents. Based on adsorption results, the biosorbent prepared from kale was the best; its maximum adsorption capacity was 93.6%, which was significantly higher than that of the lettuce biosorbent (83.7%). Characterization results indicate that different mechanisms may act simultaneously during adsorption. Non-electrostatic (hydrophobic interactions, dipole-dipole interactions, and hydrogen bonding) and electrostatic interactions (ionic attractions) together with the formation of AFB_1_-chlorophyll complexes appear to be the major influencing factors driving AFB_1_ biosorption.

## 1. Introduction

Mycotoxins are low-molecular-weight (<1 kDa) substances synthesized as part of the secondary metabolism of various fungal species, including *Aspergillus*, *Penicillium*, *Fusarium*, and *Alternaria*. Most of these secondary metabolites are extremely toxic and have significant economic implications and public health concerns. Similar to other mycotoxins, aflatoxins are a group of highly toxic metabolites that can contaminate a wide variety of food and feedstuffs. The known aflatoxins are about twenty, while the four main aflatoxins are denoted as aflatoxin B_1_ (AFB_1_), aflatoxin B_2_ (AFB_2_), aflatoxin G_1_ (AFG_1_), and aflatoxin G_2_ (AFG_2_). AFB_1_ is the most extensively studied fungal toxin because it is a potent liver carcinogen [1].

To prevent the entry of aflatoxins into the food chain, many strategies have been proposed, and although prevention is the most effective measure, many other chemical, biological, and physical methodologies have been developed [2]. Undoubtedly, the best approach to reduce aflatoxin contamination is to prevent their formation in the field; however, this is not easy. Since aflatoxins are chemically very stable molecules, almost no physical or chemical treatments can be applied without modifying the nutritional quality characteristics of the foodstuffs. Thus, the most common strategy is the inclusion of adsorbent materials into the aflatoxin-contaminated commodities to selectively bind these toxic substances during their passage through the gastrointestinal tract. For this purpose, several types of binders have been employed, which include but are not limited to: minerals (bentonite, vermiculite, montmorillonite, zeolite, hydrated sodium calcium aluminosilicate, and diatomite), chemicals (activated charcoal), polymers (polyvinylpyrrolidone, cholestyramine, chitosan, hydroxypropyl methylcellulose, sodium carboxymethylcellulose, and microcrystalline cellulose) and organics (yeast, glucomannan, lactobacilli, micronized fibres, and plant-derived sorbents) [3,4,5,6]. More elaborated adsorbents against aflatoxins have also been developed such as magnetic graphene oxide-TiO_2_ and carbon nanocomposites [7,8]. However, the high cost of some of these binders is the most critical factor that limits their applicability.

Over the last forty years, sorption with plant-derived materials has been proposed as an alternative for mycotoxin removal [9]. In this context, several studies have been conducted to evaluate the AFB_1_-sorption capacity of different agro-waste-based materials; however, to date, studies on the aflatoxin-adsorption capacity of these materials using in vitro models that mimic the physiological conditions of the gastrointestinal tract are still meager [6,10,11]. Our research group recently performed some in vitro studies with sorbents prepared from lettuce (*Lactuca sativa* L.) showing that the unmodified agro-waste-based sorbent can be successfully used at low doses for the removal of AFB_1_ [4]. Unfortunately, the used in vitro methodology may not be directly applicable to field conditions for livestock, because it does not consider the food/feed matrix effect and the enzymatic activities under in vivo-like assay conditions. Consequently, further experimental studies to investigate the adsorption potential of this and other underutilized agro-waste materials are still needed.

It is well known that one-third of the edible part of food produced becomes lost or wasted, globally, this is about 1.3 billion tons per year, including over 1 million tons of vegetable wastes [12]. As for horticultural products, the greatest waste generation occurs during the harvesting and processing stages, so it makes sense to think that horticultural wastes can be utilized to obtain value-added products. Kale (*Brassica oleracea* L.) is an important horticultural product, member of the Brassicaceae family, which possesses high contents of antioxidants and chemoprotective substances [13]. Kale is a leafy vegetal with curled leaf edges; however, accelerated leaf senescence leads to the generation of large quantities of wastes. Until today, there is currently a lack of information regarding the use of kale residues as a sorbent material for aflatoxins removal. Consequently, this research was conducted to prepare, characterize, evaluate, and compare the potential of two unmodified agro-waste-based materials for the sorption of AFB_1_ using a dynamic gastrointestinal tract-simulated model.

## 2. Results and Discussion

### 2.1. Sorption Performance

**Figure 1:**
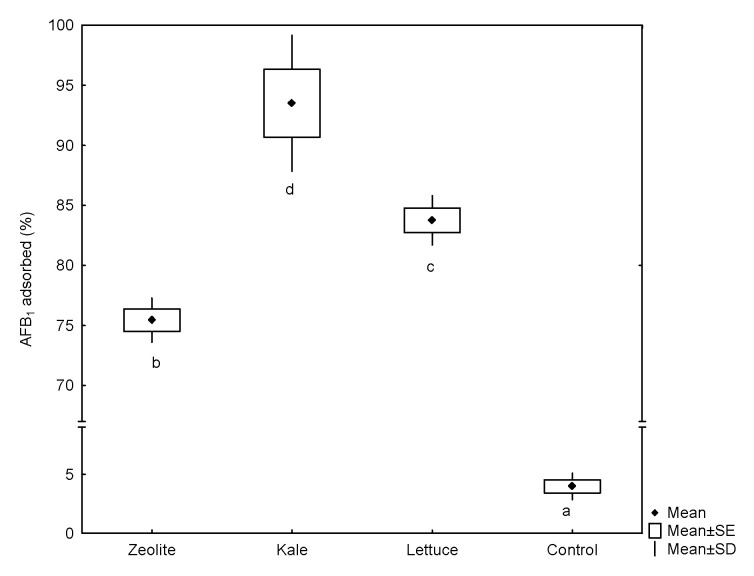
Adsorption capacities of the unmodified agro-waste-based sorbents and the inorganic mycotoxin binder (zeolite) against AFB_1_ using a dynamic gastrointestinal tract-simulated model. ^a–d^, Boxes and whiskers not sharing a common superscript differ significantly (Tukey test *p* < 0.05).

Using the dynamic gastrointestinal tract-simulated model, the sorption capacity of the tested materials—in the intestinal section—varied significantly (Figure 1). The biosorbent prepared from kale was shown to have the best adsorption performance (93.6%), followed by lettuce biosorbent (83.7%) and the inorganic mycotoxin binder (75.5%). As shown in Figure 1, the adsorption capacity of the agro-waste-based materials was significantly higher than that of the non-commercial zeolitic mineral. In contrast, controls without the addition of sorbents (reference test) show a marked lack of AFB_1_ adsorption (<4%). To date, only three studies have been conducted to evaluate the aflatoxin-sorption capacity of different plant-derived materials using static or dynamic gastrointestinal digestion procedures [6,10,11].

In this context, Adunphatcharaphon et al. [10] evaluated the aflatoxin-adsorption capacity of durian-fruit hulls (*Durio zibthinus*) using a three-step static procedure with simulated salivary, gastric, and intestinal fluids, respectively. After completing the digestion procedure (2 h), the acid-treated durian peel (0.5% *w/v*) reduced the AFB_1_ bioaccessibility by 95.1% at both gastric and intestinal levels. Moreover, Rasheed et al. [11] evaluated the adsorption capacity of blueberry pomace (0.2% *w/v*) in simulated gastric (pH 2.5) and intestinal fluids (pH 7). The removal efficiencies were about 65% and 70%, respectively. Although these studies are consistent with our findings, these methodologies may not be directly applicable to in vivo conditions because these models do not consider the food/feed matrix effect. Furthermore, the disadvantages associated with the proposed methodologies for preparing the biosorbents is that they are high energy-consuming and demand complicated procedures or highly specialized chemical substances.

Recently, our research group evaluated the AFB_1_-adsorption capacities of biosorbents prepared from the banana peel, Pyracantha leaves, and Aloe vera using a multicompartmentalized model simulating the dynamic conditions in the gastrointestinal tract [6]. In general, the organic sorptive materials (1.5%, *w/w*) significantly reduced the bioavailability of AFB_1_ in the intestinal section, being Aloe vera the biomaterial with the highest AFB_1_ adsorption capacity (68.5%). However, comparing these results with those shown in Figure 1, the adsorption capacity by these novel low-cost and bio sustainable sorbents was significantly higher than that of the Aloe vera powder, even with a less biosorbent content (0.5% *w/w*). This observation suggests that AFB_1_ adsorption by the agro-waste-based sorbents could be accomplished by different chemical and or physical mechanisms.

### 2.2. Functional Groups Involved in the Aflatoxin Adsorption

**Figure 2:**
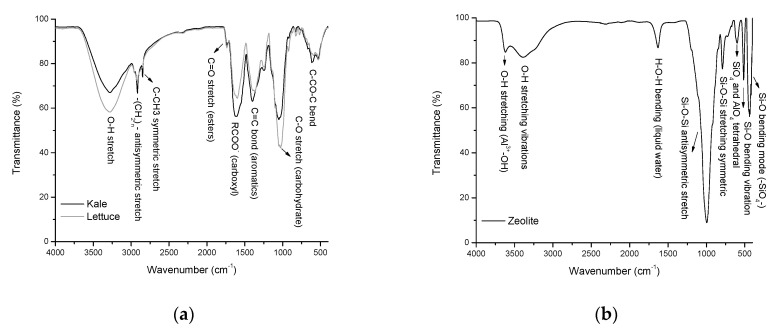
Representative Fourier-transform infrared spectra and the functional groups of (**a**) unmodified agro-waste-based sorbents and (**b**) the inorganic mycotoxin binder (zeolite).

In this research, the sorbents prepared from kale and lettuce agro-wastes adsorbed variable amounts of AFB_1_, suggesting different binding mechanisms. Therefore, sorbents were further characterized to obtain information about the interaction between the functional groups present in the sorptive materials and the AFB_1_ molecule. The experimentally measured transmittivity spectra and the functional groups of the sorptive materials via attenuated total reflection Fourier transform infrared (ATR-FTIR) spectroscopy are shown in Figure 2. Significant differences in five IR-active vibrations were detected among the biosorbents, including: (*i*) the broad frequency band related to hydroxyl groups at 3281 cm^−1^, (*ii*) the medium to weak frequency vibrations of alkyl chains at 2916 and 2850 cm^−1^, (*iii*) the strong vibration of the carboxyl group at 1613 cm^−1^, (*iv*) the strong vibration of the C=C bond in aromatics at 1406 cm^−1^, and (*v*) the strong molecular vibration of the C–O bond at approximately 1031 cm^−1^ (Figure 2, Profile a). These functional groups play essential roles in AFB_1_ adsorption, which is in close agreement with our previous works [4,6,14,15].

On the other hand, the non-commercial zeolitic mineral (Figure 2, Profile b) showed the characteristic band at around 3623 cm^−^^1^ associated with Al^3+^–OH in the octahedral sheet [16]. The zeolite was also hydrated, which was demonstrated by significant water absorption bands at 3388 and 1632 cm^−1^. The very strong band centered at 997 cm^−1^ and the medium band located at 791 cm^−1^ are related to the antisymmetric and symmetric stretch vibrations of Si–O–Si bonds. The absorption band at 598 cm^−1^ was attributed to the presence of heulandite [17]. The band at 513 cm^−1^ can be assigned to the presence of the double rings of the zeolite [18]. Finally, the absorption band at 440 cm^−1^ is associated with the bending vibration of Si–O bonds [19].

Furthermore, in the IR spectrum, the positions and numbers of the absorption bands of the agro-waste-based sorbents were essentially the same. However, significant differences in intensity (relative transmittance) were detected. In this context, band intensity and band area are two parameters commonly used to calculate a particular chemical bond concentration. Band area is often used because this parameter provides minor variation, considering that several overlapped absorption bands may occur. In this research, several chemical bonds were investigated to clarify the binding mechanisms among the biosorbents.

**Figure 3:**
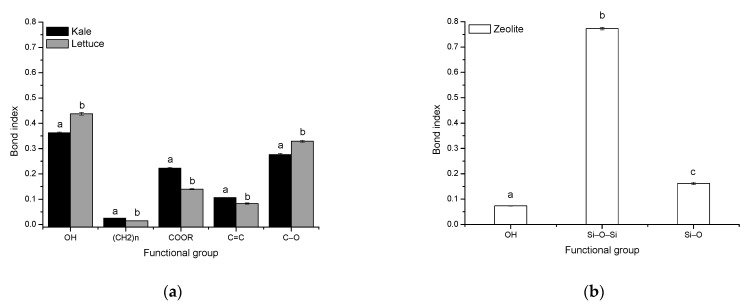
Bond indexes of the principal chemical functional groups of (**a**) unmodified agro-waste-based sorbents and (**b**) the inorganic mycotoxin binder (zeolite). Mean of five replicates ± standard error. ^a–c^, For each functional group, means not sharing a common superscript differ significantly (Tukey *p* < 0.05).

Figure 3 shows the bond indexes present in the sorptive materials. For the agro-waste-based sorbents, five bond indexes were calculated: the OH stretch, the CH_2_ and CH_3_ stretch, the C=O stretch, the C=C stretch, and the C–O stretch. In the biosorbent prepared from kale, it was found that the OH and C–O indexes were significantly lower than those in the lettuce biosorbent (Figure 3, Profile a). This decrease in OH and C–O indexes can cause low hydrophilicity in the kale biosorbent, which would make its surface more favorable to the adsorption of AFB_1_ molecules. Moreover, the CH_2_ and CH_3_, C=O, and C=C indexes were significantly higher in the kale biosorbent. The high number of hydrophobic groups may also help the kale biosorbent to remove AFB_1_ efficiently. It has been reported that an increased number of hydrophobic groups (such as methyl and aromatic groups) results in a highly hydrophobic surface, which is favorable to the adsorption capacity of sorbent materials [20].

For the non-commercial zeolitic mineral, only three bond indexes were calculated: the OH stretch, the Si–O–Si stretch and the Si–O bond (Figure 3, Profile b). In general, the bond index of Si–O–Si was significantly higher than the others, which means that the Si–O–Si surface is strictly hydrophobic [21]. Although, the AFB_1_ adsorption into clays is mediated by weak electrostatic attractions, other mechanisms such as moderate electron donor-acceptor attraction and a strong calcium-bridging linkage are also responsible for the AFB_1_ adsorption [22].

### 2.3. Point of Zero Charge (pH_pzc_) and Zeta Potential (ζ-Potential)

**Figure 4:**
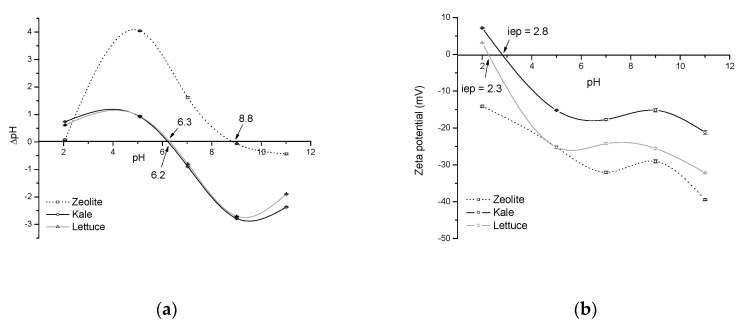
Point of zero charge (**a**), and the relationship between zeta potential and pH of the unmodified agro-waste-based sorbents and the inorganic mycotoxin binder (**b**). Mean of five replicates ± standard error. iep = isoelectric point.

The net electrical charge of the sorbent surface plays an essential role during sorption. In this regard, pH_pzc_ gives helpful information about the surface charge of the biosorbents and ensures that electrostatic interaction is one of the mechanisms that drive adsorption. In solution, at pH > pH_pzc_, the sorbent surface is negatively charged and could interact with positively charged sorbate species. In contrast, at pH < pH_pzc_, the sorbent surface becomes positively charged (the acidic water donates more protons than hydroxide groups) and starts trapping negatively charged species. Figure 4 (Profile a) shows the pH_pzc_ of the agro-waste-based sorbents and the inorganic mycotoxin binder (zeolite). It appears from this figure that the pH_pzc_, that is, the pH at which the adsorption of potential positively or negatively charged species is identical, lies in the vicinity of pH 6 for both kale and lettuce biosorbents. A pH_pzc_ near pH 9 was recorded for the zeolitic material. In this context, Nava-Ramírez et al. [4] reported a pH_pzc_ value of 5.65 for a biosorbent prepared from lettuce. Such a difference might be due in part to differences in the maturity stage, atmospheric conditions during growing, and harvest phase. Interestingly, both agro-waste-based sorbents have high negative-charged surfaces under intestinal pH simulation; as a result, these sorptive materials have significant AFB_1_ uptakes in the in vitro digestive model. On the other hand, the zeolite showed a constant positive charge in the pH range from 2 to 8.8 (Figure 4, Profile a), confirming that AFB_1_ adsorption is not driven primarily by electrostatic attractions.

To further elucidate the possible mechanism through which agro-waste-based sorbents can efficiently adsorb AFB_1_, a ζ-potential study was also conducted. Zeta potential is the electric potential in the interfacial double layer at the location of the slipping plane relative to a point in the bulk fluid away from the interface. Figure 4 (Profile b) shows the relationship between ζ-potential and pH of the sorptive materials. In general, ζ-potential significantly increased with increasing pH and reached the maximum at pH 11. It is important to highlight that both agro-waste-based sorbents presented high negative ζ-potential values in two out of three compartments of the dynamic gastrointestinal tract-simulated model; as a result, the interaction type in these compartments would be electrostatic in nature. Moreover, it was determined that the isoelectric point (iep) of the agro-waste-based sorbents occurs in the range of pH 2–3, indicating that the potential at the slipping plane (that is, the ζ-potential) is zero. In other words, at this particular pH, particles do not move when exposed to an electric field. Graph b in Figure 4 also shows that the zeolite was negatively charged throughout the entire pH range (from 2 to 11); consequently, the iep could not be determined. In general, for agro-waste-based sorbents, the iep (determined by changes in ζ-potential vs. pH) was found to be very different from the pH_pzc_ (calculated in terms of ΔpH). These differences in the iep and pH_pzc_ on the pH scale point to complex specific AFB_1_-adsorption at the interface. Interestingly, the ζ-potential values were significantly higher in the lettuce biosorbent. Under these circumstances, it would be expected that the sorbent prepared from lettuce adsorbed AFB_1_ more efficiently than the kale biosorbent. This new finding pointed out that different mechanisms could accomplish adsorption.

### 2.4. Non-Destructive Estimation of Pigments

**Figure 5:**
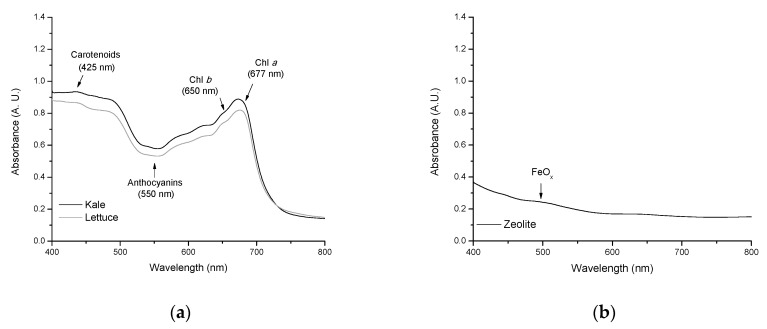
Diffuse reflectance UV-Vis spectra of (**a**) unmodified agro-waste-based sorbents and (**b**) the inorganic mycotoxin binder (zeolite).

It has been reported that pigments fulfill several important roles. For instance: chlorophylls can form strong non covalent complexes in vitro with AFB_1_ [4]; carotenoids are recognized as powerful antioxidants scavenging both singlet molecular oxygen and peroxyl radicals [23]; and anthocyanins are also potent polyphenolic antioxidants [24]. Figure 5 shows the diffuse reflectance spectra of the sorbents. For the agro-waste-based sorbents, variable absorbance was observed in both the visible and near infrared regions of the spectrum (Figure 5, Profile a). In the red region, two distinctive bands are clearly distinguished, a broad band of Chlorophyll *a* (Chl *a*) at 677 nm and a shoulder of Chlorophyll *b* (Chl *b*) at about 650 nm [6]. In the blue region—where chlorophylls and carotenoids absorb—some spectral details could be attributed to carotenoids (425 nm) and, to a lesser extent, to Chl *b* absorption [25]. Finally, in the green region, the absorbance at 550 nm can be associated with the presence of anthocyanins [26]. As shown from Figure 5 (Profile a), absorbance increases sharply with increasing pigment content (up to 0.95 A. U. in the sorbent prepared from kale). Furthermore, the zeolitic material only showed an intraconfigurational transition in the visible region near 500 nm (Figure 5, Profile b). This minor contribution is usually associated with FeO_x_ oligomers in the clay material [27].

### 2.5. Quantitative Determination of Chlorophylls and Carotenoids

**Figure 6:**
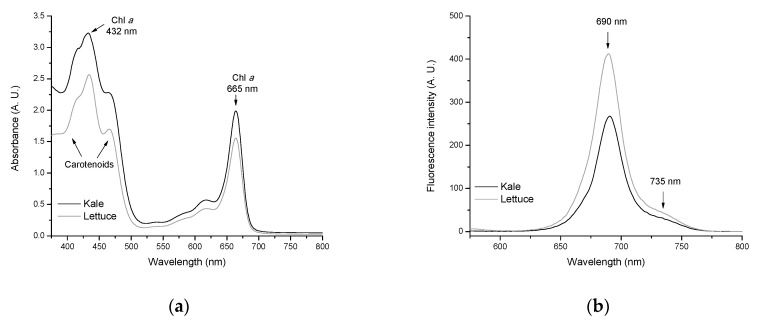
Representative chlorophyll absorption (**a**) and chlorophyll fluorescence spectra (**b**) of the unmodified agro-waste-based sorbents.

Many studies have shown that chlorophylls have significant anticarcinogenic potential against a wide range of human carcinogens, including AFB_1_ [28,29,30,31]. Therefore, different mechanisms responsible for the cancer-preventative activity have been proposed, including antioxidant activity [32,33], modulation of detoxification pathways [34], induction of apoptosis [35], and carcinogen trapping [4,36,37,38]. The last statement is more plausible because, in our previous work, it was demonstrated that the formation of AFB_1_-chlorophyll complexes improves the rate of AFB_1_ uptake by biosorbents containing considerable amounts of chlorophylls [4]. In the present study, major photosynthetic pigments were extracted with ethanol and their content was determined spectrophotometrically using the absorption coefficients of Lichtenthaler and Wellburn [39]. Figure 6 (Profile a) shows the absorption spectra of Chl *a*, Chl *b*, and total carotenoid in ethanol.

In general, the UV-Vis spectrum was dominated by the absorption of Chl *a* at 432 nm (blue region) and 665 nm (red region). Carotenoids presented a broad absorption—with three maxima—in the blue spectral range (418, 432, and 467 nm). The results obtained by UV-Vis spectroscopy indicate that the biosorbent prepared from kale had higher amounts of photosynthetic pigments in comparison with its counterpart. In an attempt to confirm these results, chlorophylls were further characterized spectrofluorometrically. Figure 6 (Profile b) shows the chlorophyll fluorescence spectra of the agro-waste-based sorbents. The spectra of both biosorbents exhibit two maxima near 690 nm and 735 nm, respectively. However, the shape of the fluorescence spectra differs considerably depending on the chlorophyll content. With increasing chlorophyll content in the biosorbent (Table 1), the fluorescence yield significantly decreased due to reabsorption of the shorter wavelength fluorescence at around 690 nm [40]. Furthermore, the ratio of chlorophyll fluorescence at the two maxima (F_690_/F_735_) as determined from the spectra of Figure 6 (Profile b) decreased considerably with increasing chlorophyll content from 0.113 (lettuce) to 0.104 (kale). These results confirmed that the biosorbent prepared from kale undoubtedly presented more chlorophyll content than lettuce biosorbent. Thus, the biosorbent prepared from kale contained approximately 30% more Chl *a* and up to 57% more total carotenoids than lettuce biosorbent (Table 1). Therefore, the hydrophobicity of chlorophylls and carotenoids leads to an improved trapping efficiency of the kale biosorbent to immobilize almost all AFB_1_ molecules in the dynamic gastrointestinal tract-simulated model.

### 2.6. The Mechanism for AFB_1_ Adsorption onto Agro-Waste-Based Sorbents

**Figure 7:**
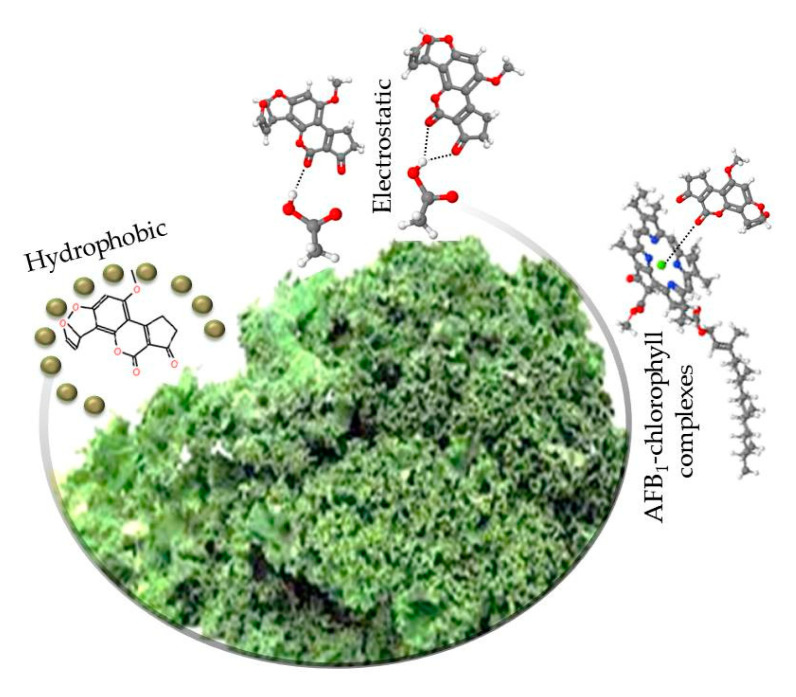
The proposed mechanism for the adsorption of AFB_1_ by the unmodified agro-waste-based materials.

The adsorption of AFB_1_ onto agro-waste-based sorbents can be a combination of electrostatic and non-electrostatic interactions (Figure 7). In general, electrostatic interactions are dependent upon the pH of the solution. At the experimental pH values of the gastrointestinal tract-simulated model (pH 2–7), the AFB_1_ molecule is neither protonated nor deprotonated. However, the adsorption of AFB_1_ was significantly increased above pH 6, suggesting that the surface of the agro-waste-based sorbents became extensively deprotonated, thereby increasing the adsorption of AFB_1_ molecules under the intestinal pH simulation (pH 7). Moreover, the agro-waste-based sorbents contain several functional groups such as hydroxyl, amino, carboxyl, and ester that can efficiently establish hydrogen bonds with the oxygen atoms of the ether, carbonyl, and methoxy groups in the AFB_1_ molecule. In particular, the pK_a_—acid dissociation constant—of a carboxylic acid is ~5; thus, it is impossible to be found in the protonated form under the experimental conditions of the intestinal section (pH 7). Consequently, the resulting carboxylate ion will not form hydrogen bonds with the oxygen atoms of the AFB_1_ molecule in this gastrointestinal tract-simulated section. Furthermore, the high number of bond indexes related to hydrophobic groups such as CH_2_, CH_3_, and C=C in the sorptive materials resulted in a highly hydrophobic surface, which was favorable to the adsorption of AFB_1_ molecules via dipole-dipole or hydrophobic interactions. Finally, the agro-waste-based sorbents also contain considerable amounts of photosynthetic pigments, among them Chl *a*, which can form strong noncovalent complexes with the AFB_1_ molecule independent of pH [4]. Consequently, the combination of these governing mechanisms resulted in materials with enhanced AFB_1_-adsorption performance.

## 3. Conclusions

In this research, two agro-waste-based sorbents were eco-friendly prepared from kale and lettuce residues and both exhibited competitive adsorption capacities. The biosorbent prepared from kale has significant potential for removing AFB_1_ in the dynamic gastrointestinal tract-simulated model (up to 93.6%). Thus, kale residues can be considered a promising agro-waste material for developing novel AFB_1_ binders. To our knowledge, this is the first report showing that unmodified agro-waste-based sorbents containing considerable amounts of photosynthetic pigments have significant ability to remove AFB_1_ in a dynamic gastrointestinal model. Although this model seems to be completer and more realistic of those available in the literature, it also has some limitations, as there are no mucosal cells, microbiota, and immune system inside the model. Consequently, biosorbents must be tested in in vivo models to demonstrate their efficacy in counteracting the toxic effects of AFB_1_ and other occurring mycotoxins such as fumonisins, ochratoxin A (OTA), and zearalenone (ZEA). Research in this direction is in progress in our laboratories.

## 4. Materials and Methods

### 4.1. Preparation of Biosorbents

#### 4.1.1. Materials

Kale (*Brassica oleracea* L.) and lettuce (*Lactuca sativa* L.) agro-wastes were kindly donated by a local horticultural producer group (Tenango del Valle, Mexico City, Mexico). Wastes were collected at the end of the winter season. A non-commercial zeolitic mineral from Taxco-Guerrero, Mexico, was used as reference material. The zeolite was powdered and sieved, and particles with diameter < 250 µm were selected to carry out the experiments.

#### 4.1.2. Unmodified Biosorbent Preparation

**Figure 8:**
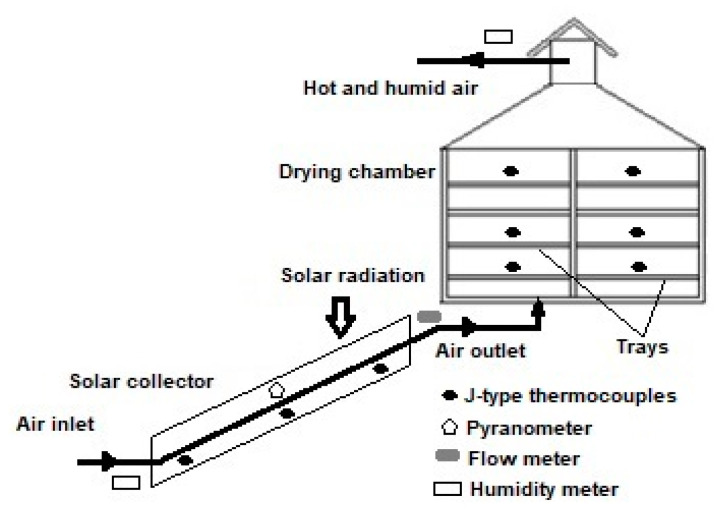
Schematic representation of the drying system.

Fresh kale and lettuce leaves were thoroughly cleaned with a brush under tap water to remove all traces of sand and soil, and then washed with distilled water. After cleaning, leaves were cut into small pieces (10 cm^2^) and separately dehydrated in a solar drying system designed and manufactured by UNAM-FES-C. As shown in Figure 8, the experimental set-up consists of a natural conventional solar dryer with a flat plate solar collector and a cabinet dryer. Drying experiments were carried out during March 2021 at the Research in Renewable Energies Laboratory (UNAM-FES-C). Kale and lettuce samples (batches of 1 kg) were uniformly distributed as a single layer on the trays and dehydrated for 10 h using a drying air temperature of 60 °C and a drying airflow rate of 0.017 kg/s. The global radiation reached the maximum value of 1800 W/m^2^ at 13:00 (local time). Weight losses of leaves were measured repeatedly until the average moisture content reached a constant value (approximately 7%). Dehydrated samples were ground and sieved (60 mesh) to obtain materials with particle sizes of <250 µm. The unmodified agro-waste-sorbent materials were stored in vacuum-sealed plastic containers at −20 °C until further analysis.

### 4.2. Adsorption Studies

#### 4.2.1. Preparation of the AFB_1_-Contaminated Diet

As a primary standard solution, aflatoxin B_1_ (100 µg AFB_1_/mL) was prepared in dimethyl sulfoxide. Afterwards, the concentrated solution was diluted to 1 µg AFB_1_/mL using distilled water. A typical maize-soybean meal diet containing 19.5% protein (13 MJ/kg metabolizable energy) was prepared. The compositional analysis of the diet is presented in Table 2. Diet samples were artificially contaminated to reach an aflatoxin content of 100 µg AFB_1_/kg. Finally, five samples were randomly taken, and the presence of AFB_1_ was confirmed using the immunoaffinity column clean-up and liquid chromatography with fluorescence detection methodology.

#### 4.2.2. Adsorption Performance

The adsorptive capacity of the different materials was evaluated in a dynamic gastrointestinal tract-simulated model reported by Hernandez-Patlán et al. [41] with minimal modifications. The assay was performed with one control (zeolite) and two different treatments (agro-waste-based sorbents). The experimental set-up consists of a biochemical oxygen demand incubator set at 40 °C accessorized with an orbital shaker. Tubes were held at 30° angle inclination to enable proper blending. Briefly, 5 g of the aflatoxin-contaminated feed (100 µg AFB_1_/kg) plus 0.5% (*w/w*) sorbent were placed in 50 mL polypropylene centrifuge tubes and mixed vigorously. To simulate the enlarged part of the esophagus environment, tubes were added with 10 mL of 0.03 M HCl, reaching values around pH 5. All tubes were incubated for 30 min at 40 °C and shaken at 19 rpm. After the incubation, in each tube, 2.5 mL of 1.5 M HCl and 3000 U of pepsin (Merck KGaA, Darmstadt, Germany) per gram of diet were added to reach a pH of around 2. These conditions simulated the beginning of digestion, and tubes were incubated again 45 min. The third step was intended to mimic the intestinal section (pH ~ 7). For that, 6.84 mg of 8× pancreatin (Merck KGaA, Darmstadt, Germany) in 6.5 mL of 1 M NaHCO_3_ were added and tubes were incubated for another 120 min. Under these circumstances, the complete in vitro digestion procedure took 195 min. At the end of the incubation, tubes were centrifuged at 7000× *g* for 30 min and the supernatant was collected for AFB_1_ quantification. Control samples (without adsorbents) were used to know the real concentration of AFB_1_ per tube under the gastrointestinal tract-simulated conditions. All determinations were carried out in quintuplicate.

### 4.3. Analytical Methods

#### 4.3.1. Aflatoxin Assay

The supernatant was cleaned up with monoclonal antibody-based immunoaffinity columns (Vicam, Watertown, MA, USA), and the eluate obtained was used for ultra-performance liquid chromatography (UPLC) analysis. A modified method previously described by Hernández-Ramírez et al. [42] was employed. An ultra-performance liquid chromatograph system (Waters ACQUITY H-class) equipped with a quaternary solvent manager and a reverse phase column (2.1 mm × 100 mm, 1.7 µm particles) was used. Methanolic extracts collected from the immunoaffinity columns (1 µL) were injected and eluted with a mobile phase of water:methanol:acetonitrile (64:18:18) at a flow rate of 0.7 mL/min. Detection was accomplished with a fluorescence detector set at 365 nm excitation and 429 nm emission. The AFB_1_ concentration was calculated using a standard reference (AFB_1_, Merck KGaA, Darmstadt, Germany) with a calibration curve. The detection limit of AFB_1_ was found to be 0.002 µg/L.

#### 4.3.2. ATR-FTIR Spectroscopy

In this study, ATR-FTIR spectroscopy was used to analyze the vibrational features of the sorbents. The spectra were acquired using a Frontier SP8000 FTIR spectrophotometer (Perkin Elmer, Waltham, MA, USA) in the wavenumber range 4000–400 cm^−1^ at a resolution of 4 cm^−1^. The spectra were subsequently analyzed with the Spectrum 10.4.2 software. The bond indexes (BI) of the principal chemical functional groups were calculated by using the following equations:

- For the agro-waste-based sorbents:
  (1) $${\mathrm{BI}}_{\mathrm{OH}}=\frac{{\mathrm{BA}}_{3281}}{\sum \;\mathrm{BA}}$$
  (2) $${\mathrm{BI}}_{\left({\mathrm{CH}}_{2}\right)n}=\frac{{\mathrm{BA}}_{2916}+{\mathrm{BA}}_{2850}}{\sum \;\mathrm{BA}}$$
  (3) $${\mathrm{BI}}_{\mathrm{COOR}}=\frac{{\mathrm{BA}}_{1613}}{\sum \;\mathrm{BA}}$$
  (4) $${\mathrm{BI}}_{C=C}=\frac{{\mathrm{BA}}_{1406}}{\sum \;\mathrm{BA}}$$
  (5) $${\mathrm{BI}}_{C-O}=\frac{{\mathrm{BA}}_{1031}}{\sum \;\mathrm{BA}}$$
- For the zeolitic mineral:
  (6) $${\mathrm{BI}}_{\mathrm{OH}}=\frac{{\mathrm{BA}}_{3623}+{\mathrm{BA}}_{3388}+{\mathrm{BA}}_{3623}}{\sum \;\mathrm{BA}}$$
  (7) $${\mathrm{BI}}_{\mathrm{Si}-O-\mathrm{Si}}=\frac{{\mathrm{BA}}_{997}+{\mathrm{BA}}_{791}}{\sum \;\mathrm{BA}}$$
  (8) $${\mathrm{BI}}_{\mathrm{Si}-O}=\frac{{\mathrm{BA}}_{598}+{\mathrm{BA}}_{513}+{\mathrm{BA}}_{440}}{\sum \;\mathrm{BA}}$$
  where BA_x_ = the band area around the corresponding wavenumber (cm^−1^), and Σ BA = the total area of all bands in the corresponding IR spectrum.

#### 4.3.3. Point of Zero Charge (pH_pzc_) and Zeta Potential (ζ-Potential) Measurements

The pH_pzc_ methodology was performed by adding identical amounts of sorbents (125 mg) to a set of flasks containing distilled water at different pH values (2, 5, 7, 9, and 11). The pH was adjusted with HCl (0.1 M) or NaOH (0.1 M) as needed. The pH values of the distilled water were denoted as the initial pH (pH_i_). Subsequently, samples were shaken for 195 min using an orbital shaker at 200 rpm. After settling, the pH value of the supernatant was measured using a combination glass electrode and denoted as the final pH (pH_f_). The pH_pzc_ was obtained from the plot of ΔpH (pH_f_-pH_i_) against pH_i_. Furthermore, ζ-potential measurements were performed with a ZetaSizer Pro (Malvern Instruments, Worcestershire, UK). All determinations were performed at room temperature by diluting 500 μL of the sorbent suspension (0.5% *w/v*) in 5 mL deionized water. Samples were evaluated at five different pH values (2, 5, 7, 9, and 11), including those that simulate the multicompartmentalized gastrointestinal model. The isoelectric point (iep) defined as the pH at which the ζ-potential was zero was obtained by plotting the curve of ζ-potential against pH. Each set of experiments (pH_pzc_ and ζ-potential) was performed in quintuplicate.

#### 4.3.4. Determination of Chlorophylls and Carotenoids in the Agro-Waste-Based Sorbents

##### Spectral Reflectance Measurements

Diffuse reflectance spectra were recorded in a range of 400–800 nm with a Lambda 365 UV-Vis spectrophotometer (Perkin Elmer, Waltham, MA, USA) equipped with a 100 mm integrating sphere attachment to capture diffusely reflected light. To provide 100% reflectance, barium sulfate (BaSO_4_) was used as reference material. Spectral data were interfaced to a personal computer for further processing using the UV Lab software (Perkin Elmer, Waltham, MA, USA).

##### Photosynthetic Pigment Analysis

The pigments were quantitatively determined in the samples used for collecting the diffuse reflectance spectra. Chlorophylls and carotenoids were extracted with ethanol and determined spectrophotometrically using a Cary 8454 UV-Vis Diode Array System spectrophotometer (Agilent Technologies, Santa Clara, CA, USA). The absorption coefficients reported by Lichtenthaler and Wellburn [39] were used for Chl *a*, Chl *b*, and total carotenoid (C *x* + *c*) estimation. The following equations were utilized to determine their contents:

(9) $$\mathrm{Chl}\;a=13.95\;A_{665}-6.88\;A_{649}$$

(10) $$\mathrm{Chl}\;b=24.96\;A_{649}-7.32\;A_{665}$$

(11) $$C\;x+c_{}=\frac{1000\;A_{470}-2.05\;\mathrm{Chl}\;a-114.8\;\mathrm{Chl}\;b}{245}$$

To further characterize chlorophylls in the biosorbents, the ethanolic extracts were also subjected to spectrofluorometry. For this purpose, fluorescence measurements were performed using a fluorescence LS-55 spectrophotometer (Perkin Elmer, Waltham, MA, USA). Spectra were recorded in the wavelength range of 575–800 nm using a 1 cm path quartz cell. The fluorescence spectra were collected at an excitation wavelength of 440 nm using equally wide excitation and emission slits (5 nm). The ratio of chlorophyll fluorescence at the two maxima (F_690_/F_735_) was used to indicate the potential photosynthetic activity.

### 4.4. Experimental Design and Statistical Analysis

The experiment was conducted as a completely randomized design with five replicates. Experimental data were subjected to one-way analysis of variance (ANOVA), and means were separated using the Tukey procedure with the SAS software [43]. A significance value of α = 0.05 was used to distinguish significant differences between treatments.

## Figures and Tables

**Table 1:** Chlorophylls and total carotenoid contents of the unmodified agro-waste-based sorbents.

Photosynthetic Pigment (µg/g)	Kale	Lettuce
Chlorophyll *a*	4148.6 ± 137 ^a^	3224.1 ± 99 ^b^
Chlorophyll *b*	2178.5 ± 43 ^a^	1809.6 ± 54 ^b^
Total chlorophyll (*a* + *b*)	6327.1 ± 152 ^a^	5033.7 ± 128 ^b^
Total carotenoid (*x* + *c*) ^1^	796.5 ± 25 ^a^	506.1 ± 16 ^b^

^1^*x* + *c* = xantophyll + carotenes. Mean of five replicates ± standard error. ^a,b^, Means, within the same row, not sharing a common superscript differ significantly (Tukey test *p* < 0.05).

**Table 2:** The compositional analysis of the diet.

Ingredient	%
Maize	57.45
Soybean meal	34.66
Vegetable oil	3.45
Dicalcium phosphate	1.86
Calcium carbonate	0.99
Sodium chloride	0.38
Vitamin premix ^1^	0.10
Mineral premix ^2^	0.10
DL-Methionine	0.33
Choline chloride (60%)	0.20
Antioxidant (ethoxyquin)	0.05
L-Lysine HCl	0.31
Threonine	0.12

^1^ Vitamin premix supplied the following per kg: vitamin A, 20,000,000 IU; vitamin D3, 6,000,000 IU; vitamin E, 75,000 IU; vitamin K3, 9 mg; thiamine, 3 mg; riboflavin, 8 mg; pantothenic acid, 18 mg; niacin, 60 mg; pyridoxine, 5 mg; folic acid, 2 mg; biotin, 0.2 mg; cyanocobalamin, 16 mg; and ascorbic acid, 200 mg. ^2^ Mineral premix supplied the following per kg: Mn, 120 mg; Zn, 100 mg; Fe, 120 mg; Cu, 10–15 mg; I, 0.7 mg; Se, 0.4 mg; and Co, 0.2 mg.
